# Supplementary material for: Crawling and Gliding: A Computational Model for Shape-Driven Cell Migration
Source: PLoS Comput Biol. 2015 Oct 21;11(10):e1004280. doi: 10.1371/journal.pcbi.1004280 (PMC4619082; doi:10.1371/journal.pcbi.1004280)
Supplement: S1 Code — (ZIP) [file pcbi.1004280.s012.zip › release/tst/doc/html/qtgraph_8cpp.html]

Tissue Simulation Toolkit: qtgraph.cpp File Reference


Tissue Simulation Toolkit
 0.1.4.1


- Main Page
- Namespaces
- Classes
- Files

- File List
- File Members

qtgraph.cpp File Reference

`#include <qapplication.h>`  
`#include <qwidget.h>`  
`#include <qlabel.h>`  
`#include <qpainter.h>`  
`#include <q3picture.h>`  
`#include <QPalette>`  
`#include <QMouseEvent>`  
`#include <QPaintEvent>`  
`#include <QImageWriter>`  
`#include <malloc.h>`  
`#include <iostream>`  
`#include <qtimer.h>`  
`#include <qpixmap.h>`  
`#include <qimage.h>`  
`#include <q3strlist.h>`  
`#include <QResizeEvent>`  
`#include "qtgraph.h"`  
`#include "parameter.h"`

Include dependency graph for qtgraph.cpp:


---

Generated on Thu Aug 14 2014 22:04:01 for Tissue Simulation Toolkit by  

 1.8.6
